# The Relevance of Testing the Efficacy of Anti-Angiogenesis Treatments on Cells Derived from Primary Tumors: A New Method for the Personalized Treatment of Renal Cell Carcinoma

**DOI:** 10.1371/journal.pone.0089449

**Published:** 2014-03-27

**Authors:** Renaud Grépin, Damien Ambrosetti, Alexandre Marsaud, Lauris Gastaud, Jean Amiel, Florence Pedeutour, Gilles Pagès

**Affiliations:** 1 Biomedical Research Unit, Centre Scientifique of Monaco, Principality of Monaco; 2 Institute for Research on Cancer and Aging of Nice (IRCAN) UMR/7284 U1081, Nice University Hospital, Central Laboratory of Pathology, University of Nice Sophia Antipolis, Nice, France; 3 Institute for Research on Cancer and Aging of Nice (IRCAN) UMR/7284 U1081, Nice University Hospital, Department of Urology, University of Nice Sophia Antipolis, Nice, France; 4 Department of Medical Oncology, Centre Antoine Lacassagne, Nice, France; 5 Nice University Hospital, Department of Urology, University of Nice Sophia Antipolis, Nice, France; 6 Institute for Research on Cancer and Aging of Nice (IRCAN) UMR/7284 U1081, Nice University Hospital, Laboratory of Solid Tumors Genetics, University of Nice Sophia Antipolis, Nice, France; 7 Institute for Research on Cancer and Aging of Nice (IRCAN) UMR/7284 U1081, University of Nice Sophia Antipolis, Nice, France; University of Kentucky College of Medicine, United States of America

## Abstract

Despite the numerous available drugs, the most appropriate treatments for patients affected by common or rare renal cell carcinomas (RCC), like those associated with the Xp11.2 translocation/transcription factor for immunoglobulin heavy-chain enhancer 3 (*TFE3*) gene fusion (TFE3 RCC), are not clearly defined. We aimed to make a parallel between the sensitivity to targeted therapies on living patients and on cells derived from the initial tumor. Three patients diagnosed with a metastatic RCC (one clear cell RCC [ccRCC], two TFE3 RCC) were treated with anti-angiogenesis drugs. The concentrations of the different drugs giving 50% inhibition of cell proliferation (IC50) were determined with the Thiazolyl Blue Tetrazolium Bromide (MTT) assay on cells from the primary tumors and a reference sensitive RCC cell line (786-O). We considered the cells to be sensitive if the IC50 was lower or equal to that in 786-O cells, and insensitive if the IC50 was higher to that in 786-O cells (IC 50 of 6±1 µM for sunitinib, 10±1 µM for everolimus and 6±1 µM for sorafenib). Based on this standard, the response in patients and in cells was equivalent. The efficacy of anti-angiogenesis therapies was also tested in cells obtained from five patients with non-metastatic ccRCC, and untreated as recommended by clinical practice in order to determine the best treatment in case of progression toward a metastatic grade. *In vitro* experiments may represent a method for evaluating the best first-line treatment for personalized management of ccRCC during the period following surgery.

## Introduction

Even though ccRCC represent the most common type of kidney cancer, approximately 20% of RCC are not ccRCC [1]. Representative examples of rare RCC include those associated with Xp11.2 translocation which leads to the rearrangement of the transcription factor E3 (*TFE3*) gene [2]. *TFE3* fuses with any of the following gene partners; papillary renal cell carcinomas (PRCC) [3], non-POU domain containing octamer binding (*NONO*), splicing factor proline/glutamine rich (PFS) [4], renal cell carcinoma chromosome 17 (RCC 17) [5], clathrin heavy chain (CLTC) [6] and an unknown gene present on chromosome 19 [7]. *TFE3* RCC presents both papillary/alveolar architecture and tumor cells with a clear cytoplasm. Tumors characterized with chromosomal translocations involving the *TFE3* gene show strong nuclear immuno-reactivity of the TFE3 protein. Over-expression of the fusion protein induces abnormal cell proliferation and motility as a result of its binding to the E2F3 transcription factor [8], impaired binding of the checkpoint protein MAD2B [9], deregulation of cell-cycle mediators [10]–[11] and expression of the c-MET tyrosine kinase receptor [12].

The gold standard treatments for metastatic ccRCC are currently sunitinib (inhibitor of tyrosine kinase receptors including the colony stimulating factor 1 receptor (CSF-1R), the fms-like tyrosine kinase 3 receptor (FLT-3), the stem cell factor receptor (c-KIT), the platelet-derived growth factor receptor (PDGF-R), the receptor for glial cell line-derived neurotrophic factor family, “rearranged during transfection” (RET) and the vascular endothelial growth factor receptors 1, 2, 3 (VEGF-R1, 2, 3), sorafenib (inhibitor of the same receptors and B and c-RAF) and : temsirolimus/everolimus (inhibitor of mammalian target of rapamycin (mTOR).

In Xp11 translocation RCC, anti-angiogenesis drugs give similar results in terms of objective responses and prolonged progression free survival to those reported for ccRCC [13].

Whereas some patients clearly benefit from their treatment, others are totally refractory due to the acquisition of resistant cell populations [14]. Moreover, some adverse events have been described [15].

Hence, for both ccRCC and non-ccRCC, physicians need a rapid method to determine the best therapy considering the poor prognosis of these cancers in the metastatic phase. We derived cells from the tumors of three patients; one diagnosed with a ccRCC and two with TFE3 RCC and assessed their sensitivity to different anti-angiogenesis drugs. The *in vitro* sensitivity to these drugs was tested on non-metastatic ccRCC in order to determine the best treatment in case of progression towards a metastatic grade.

## Patients and Materials and Methods

### Patients

**Figure 1:**
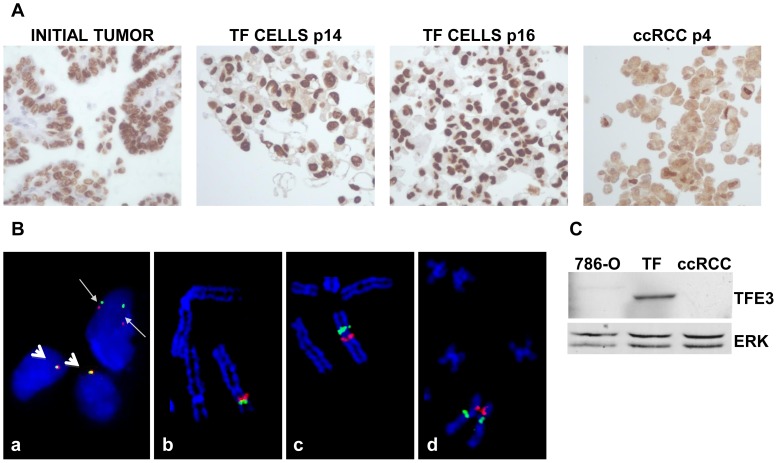
Characterization of TFE3 expression and *TFE3* rearrangement in the initial tumor and in TF cells. A) Immunohistochemical staining for TFE3 of the initial tumor. Labeling with anti-TFE3 antibodies was also performed on cells from passages 14 and 16 (P14 and P16 TFE3 cells) embedded in paraffin. TFE3 labeling was also performed on ccRCC cells cultured under the same conditions as TF cells. ccRCC cells served as a negative control. Note the cytoplasmic background instead of only nuclear labeling. B) **Image a**: An uncultured cell suspension from the renal cell tumor hybridized with a dual-color break-apart FISH probe framing *TFE3*. A rearrangement of *TFE3* in the upper nucleus (tumor cell) is observed with BAC probes CTD-2534B7 (red signal; 3′ side of *TFE3*) and CTD-3009K20 (green signal; 5′ side of TFE3). The red and green signals are clearly separated as a result of the pericentric inversion of chromosome X (thin arrows). Two rearranged signals are observed because the abnormal chromosome X is duplicated in tumor cells. In contrast, red and green signals are closely juxtaposed in the two normal nuclei that contain one X chromosome, respectively (thick arrows). **Image b**: A normal partial metaphase cell hybridized with a dual color break-apart BAC FISH probe framing *TFE3*. BAC probes CTD-2534B7 (red signal; 3′ side of *TFE3*) and CTD-3009K20 (green signal; 5′ side of *TFE3*) are closely juxtaposed at Xp11.23 on the short arm of the X chromosome. **Image c**: A partial abnormal tumor metaphase cell (cell line, passage 9) hybridized with a dual color break-apart FISH probe framing *TFE3*. As a result of the X chromosome pericentric inversion, BAC probe CTD-3009K20 (green signal; 5′ side of *TFE3*) is translocated from its normal location at Xp11.23 to *NONO* locus at Xq13.1 on the long arm of the X chromosome. BAC probes CTD-2534B7 (red signal; 3′ side of *TFE3*) remains at the original *TFE3* locus at Xp11.23. **Image d**: A partial abnormal tumor metaphase cell (cell line, passage 9) hybridized with a dual-color break-apart FISH probe framing *NONO*. As a result of the X chromosome pericentric inversion, BAC probe RP11-753F2 (green signal; 5′ side of *NONO*) is translocated from its normal location at Xq13.1 to *TFE3* locus at Xp11.23 on the short arm of the X chromosome. BAC probes RP11-624G23 (red signal; 3′ side of *NONO*) remains at the original *NONO* locus at Xq13.1. C) Western blot analysis of the presence of TFE3 in cells from the “TFE3” tumor, in ccRCC 786-O cells and ccRCC cells obtained from an independent tumor. 786-O and ccRCC cells served as negative controls. ERK served as a loading control.

The Ethic departments of the University hospital and of the Cancer centre (Centre Antoine Lacassagne), Nice, FRANCE specifically approved this study. Participants provide their written informed consent to participate in this study and to publish these case details according to our institutional ethics rules. Bone, lung or liver metastasis was confirmed for three RCC patients by magnetic resonance imaging. For the first and the third patient, the pathology report indicated a Fuhrman grade 3, pT3a ccRCC. FISH and immunohistochemistry confirmed Xp11.2 translocation, the presence of a *TFE3*-*NONO* fusion and over-expression of the fusion protein (TF RCC, Fig. 1A, 1B, 1C). The second patient had a Fuhrman grade 4, pT3a ccRCC with a chromosome 3p deletion, subsequent loss of von Hippel Lindau gene (*vhl*), a chromosome 5q gain and other genetic anomalies. The clinical/genetic characteristics of additional five ccRCC patients are shown in Table 1.

**Table 1:** Clinical and genetic characteristics of the metastatic and non-metastatic patients.

Name	Fuhrman Grade	TNM	Clinical description
TF	3	pT3a	Metastatic (bones), Xp11.2 translocation, TFE3-NONO fusion
M	3	pT3a	Metastatic (lung and liver), Xp11.2 translocation, TFE3-NONO fusion
CC	4	pT3a	Metastatic (bones), chromosome 3p deletion, loss of von Hippel Lindau gene, chromosome 5q gain, and other genetic anomalies
2a	2	pT3b	Non metastatic, monosomy 3
2b	2	pT1b	Non metastatic, 3p deletion loss of von Hippel Lindau gene, trisomy 5q
3a	3	pT3a	Non metastatic, monosomy 3, trisomy 5
4a	4	pT3a	Non metastatic, monosomy 3, trisomy 5, monosomy 14
4b	4	pT3a	Non metastatic, 3p deletion loss of VHL, trisomy 5, monosomy 14

Metastatic patients are presented on a black background; non metastatic patients are presented on a grey background.

The first TFE3 RCC patient was treated with 37.5 mg sunitinib per day for 6 weeks the dose was increased to 50 mg per day by the seventh week. Computed tomography scanning (CT) showed regression of the tumor and of the lymphatic nodes 5 weeks after the start of the anti-angiogenesis treatment. Left nephrectomy with lymphadenectomy was performed in April 2011 after a 30-day interruption in sunitinib treatment. One month after surgery, a CT scan showed lymph node progression. Sunitinib treatment at 50 mg per day was resumed for a second cycle of 4 weeks. In August 2011, a CT scan revealed an increasing size of the pelvic adenopathy and a pathological examination of a tissue biopsy confirmed lymph node metastasis. Sunitinib treatment was stopped and replaced by treatment with everolimus. A CT scan performed in November 2011 revealed lymphadenopathy with progression into the mediastinum, abdomen and pelvis, in addition to an increase in bone lesions. Everolimus was replaced by sorafenib (400 mg twice a day). A CT scan performed in January 2012 showed stability of the lesions. Sorafenib treatment was continued until the patient's death on June 2012 following a rapid deterioration in physical status despite stability of the lesions.

For the ccRCC patient, right nephrectomy was performed in December 2011. Sunitinib treatment at 50 mg per day was started in January 2012. In November 2012, the patient was stable and sunitinib treatment was stopped. Progression was observed in May 2013. The patient was treated with everolimus in May 2013. A CT scan in July 2013 revealed stability of the lesions but progression was observed in October 2013. The patient was placed in palliative care.

For the second TFE3 RCC patient, sunitinib treatment was started in April 2012. The patient was responsive until October 2012. A specific ascite developed in mid-November 2012. The patient received anti-mTOR treatment and was responsive for 1 month and then died following deterioration in physical status despite stability of the lesions.

### Cell culture

Tumor fragments were treated with collagenase overnight at 37°C and/or mechanically disaggregated with scalpels. The second patient's TFE3 tumor was necrotic and we did not succeed in obtaining cells from the primary tumor. However, we were able to obtain cells from the adjacent invaded tissues (kidney vein, adrenal gland and lymph nodes). Tumor cells (TF, cells from the first TFE3 patient, M cells from invaded tissue of the second TFE3 patient, CC cells from the ccRCC patient) were suspended in cell culture medium specific for renal cells (PromoCell, Heidelberg Germany). The FISH, immunohistochemical and Western blot analyses of the initial tumor and cultured tumor cells, even after many passages, confirmed the presence of a *TFE3*-*NONO* fusion and over-expression of the fusion protein (Fig. 1A, 1B, 1C). The 786-O cells were from the American Type Culture Collection (ATCC) and cultured in the same defined medium.

### PCR experiments

Quantitative PCR (qPCR) experiments, semi quantitative PCR (loading on agarose gel stained with ethidium bromide and quantification with the program Image J) were also performed after cell passage 11. One microgram of total RNA was used for reverse transcription, using the QuantiTect Reverse Transcription kit (QIAGEN, Hilden, Germany), with blend of oligo(dT) and random primers to prime first-strand synthesis. For real-time PCR, we used the master mix plus for SYBR assays (Eurogentec, Liege, Belgium). To calculate the relative expression of the different mRNAs, the 2^[−ΔΔC(T)]^ method was used [16]. The PCR conditions were 10 minutes at 95°c followed by 40 cycles 15 seconds at 95°C, 1 minute at 60°C.

The following oligos were used: CSF-1R: Forward; 5′- TCCAAAACACGGGGACCTATC-3′; Reverse: 5′- CGGGCAGGGTCTTTGACATA-3′; KIT: Forward: ; 5′- CGTTCTGCTCCTACTGCTTCG-3′: Reverse: 5′- CCACGCGGACTATTAAGTCTGA-3′; FLT3: Forward: 5′- CGGGCTCACCTGGGAATTAG-3′; Reverse: 5′- GTCGTTTCTTGCCACTGATGA-3′; PDGFR beta: Forward: 5′- AGCACCTTCGTTCTGACCTG-3′; Reverse: 5′- TATTCTCCCGTGTCTAGCCCA-3′; VEGFR2: Forward: 5′- GTGATCGGAAATGACACTGGAG-3′; Reverse: 5′- CATGTTGGTCACTAACAGAAGCA-3′; HGF: Forward: 5′-GCTATCGGGGTAAAGACCTACA-3′; Reverse: 5′-CGTAGCGTACCTCTGGATTGC-3′; MET: Forward : Reverse: 5′-GCAGTGAACCTCCGACTGTATG-3′; NRP2: Forward:5′- GCTGGCTATATCACCTCTCCC-3′: Reverse: 5′-TCTCGATTTCAAAGTGAGGGTTG-3′; NRP1: Forward: 5′-GGCGCTTTTCGCAACGATAAA-3′: Reverse: 5′-TCGCATTTTTCACTTGGGTGAT-3′; CXCL7: Forward: 5′-TGGCGAAAGGCAAAGAGGAAAGTC-3′: Reverse: 5′- TTGGTTGCAATGGGTTCCTTTCCC-3′; CXCL8: Forward: 5′-ATG ACT TCC AAG CTG GCC GT-3′: Reverse: 5′-TCC TTG GCA AAA CTG CAC CT-3′. After completion of the qPCR reaction, amplified cDNAs were loaded on agarose gels and stained with ethidium bromide.

### Antibodies

The following antibodies were used for Western blotting: anti-phospho ERK 1,2 (Sigma St Louis, MO, Reference M8159)), anti-phospho Akt, anti-Akt and anti-phospho S6 Kinase (Cell Signaling, Cambridge, UK, respectively References 4051, 9272, 9234) and anti-TFE3 anti ERKs (Santa Cruz Biotechnology, Santa Cruz, CA references sc 5958 (TFE3) and sc 93 (ERK)).

### Immunohistochemistry

Cell pellets were fixed in phosphate buffered formaldehyde, then dehydrated through a series of ethanol concentrations, cleared with xylene, embedded in paraffin and 5 µm sections were prepared. Tumor sections (3–4 µm) were handled as described previously [17]. Briefly, Sections were deparaffinized in two changes of toluene and hydrated through graded alcohol and water. Sections were rinsed with phosphate buffer saline (PBS). Antigen retrieval was performed using proteinase K solution (5 min). Endogenous peroxidase activity was blocked with 3% H2O2 (5 min). Sections were then incubated with with mouse monoclonal anti-TFE3 antibodies for 30 min at room temperature and rinsed with PBS. Revelation was performed using the Envision System according to the manufacturer's recommendations, with diaminobenzidine as chromogen. Sections were counterstained with hematoxylin. Cell pellets or experimental tumors were fixed in phosphate buffered formaldehyde, then dehydrated through a series of ethanol concentrations, cleared with xylene, embedded in paraffin and 5 µm sections were prepared. For histological examination, the sections were stained using Haematoxylin–Erythrosine–Safran (HES) for morphology analysis and alcyan blue for proteoglycan detection. Indirect immunostaining assay was performed as described previously [17].

### Tumor xenograft formation and size evaluation

This study was carried out in strict accordance with the recommendations in the Guide for the Care and Use of Laboratory Animals of Centre National de la Recherche Scientifique (CNRS). All efforts were made to minimize suffering. Our experiments were approved by the “Comité national institutionnel d'éthique pour l'animal de laboratoire (CIEPAL)”. The project is registered under the reference: NCE/2013-97. The mice were housed under strict pathogen-free conditions and given sterile food and water. Animals were housed 5 per cage. 10^7^ cells were injected subcutaneously into the flank of 5-week-old nude (nu/nu) female mice (Janvier, France) under sodium pentobarbital anesthesia. The tumor volume was determined by using a caliper (v = L×l^2^×0.52 [18]). When the tumor reached 100 mm3, mice were treated by daily gavage for fifteen days with placebo (dextrose-water vehicle), sunitinib (40 mg/kg) [19] or sorafenib (80 mg/kg) [20]. The ratio between the size of the tumor before and at the end of treatment for each tumor was measured.

## Results

### Expression of the receptors targeted by sunitinib and sorafenib on tumor cells

Considering that the effects of anti-angiogenesis treatments on endothelial cells are equivalent for any patient, the different responses to the drugs may be attributed to the genetic variability of tumor cells expressing the target receptors and/or over-activating the mTOR pathway. Moreover, sunitinib and sorafenib inhibit vascular endothelial cell and tumor cell proliferation because both cell types express target receptors but to a greater extent than normal renal epithelial cells [21]. Hence, we analyzed the expression of tyrosine kinase receptors targeted by sunitinib and sorafenib in cells obtained from tumor tissues and on the 786-O cells. The relative expression levels of these receptors were determined by qPCR and confirmed by semi-quantitative PCR. 786-O cells do not express FLT-3, c-KIT or VEGF-R2, TF cells express all the receptors except VEGF-R2, CC cells express all the receptors except VEGF-R1, and M cells express all the receptors except FLT-3. The expression score (number of expressed receptors and relative levels) given in Table 2 showed that M cells expressed the receptors at the highest level and 786-O cells had the lowest level of receptor expression (786-O cells<CC cells<TF cells<M cells).

**Table 2:** Level of expression of the different receptors targeted by sunitinib and sorafenib.

	CSFR	FLT3	KIT	PDGFR	RET	VEGFR1	VEGFR2	VEGFR3	Score/8	E Score
786-O	100	100	100	100	100	100	100	100		800
	+	+	−	+	+	−	−	+	5	
ccRCC	2	68	1284	139	100	112	5198	147		7050
	+	+	+	+	+	−	+	+	7	
Met	6	145	11039	332	50	3274	1297	128		16271
	+	+	+	+	+	+	+	+	8	

(−) indicates that no or very low levels of detectable amplified cDNA were observed on an agarose gel; (+) indicates amplified cDNA. The *Score/8* indicates the presence of receptorsargeted by sunitinib or sorafenib. The *E Score* indicates the relative expression level as detected by quantitative and semi-quantitative PCR. A level of sensitivity to sunitinib similar to that of 786-O cells is indicated by a grey background; sensitivity only to higher concentrations of the drug is indicated by a black background.

### Determination of the *in vitro* sensitivity to the drugs: correlation with the patients' response

**Figure 2:**
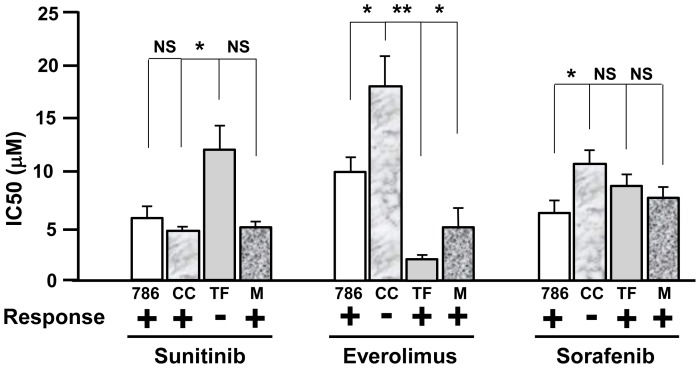
*In vitro* sensitivity of the TF, CC and M cells to targeted therapies. The IC50 of the different drugs was determined using the MTT test with concentrations of the drugs ranging from 0.1 µM to 100 µM in 786-O (786), TF, CC and M cells. The means SEMs and p-values (Student t test), in comparison with the IC50 for 786-O cells, the reference value, are shown. *p<0.05; **p<0.01 (Student t test).

To establish a correlation between the therapeutic response observed in the patients and response of the cells derived from the tumors, we performed *in vitro* proliferation assays in the presence of increasing concentrations of the different drugs used to treat the patients (from 0.1 µM to 100 µM). We used 786-O cells as a reference since their proliferation is inhibited by sunitinib [22]–[23], sorafenib [24]–[25] and mTOR inhibitor [26]. We considered the cells to be sensitive to a particular drug if the concentration giving 50% inhibition of cell proliferation (IC50) was lower than or equal to the IC50 in 786-O cells and considered to be resistant if the IC50 was higher than in 786-O cells. The IC50 of 786-O cells for sunitinib, everolimus and sorafenib were respectively 6±1, 10±1 and 6±1 µM. Following 48 hours of treatment the IC50 for sunitinib was equivalent for 786-O, CC and M cells and higher for TF cells (Fig. 2). These results showed that TF cells were resistant to sunitinib whereas CC and M cells were sensitive, a result that correlated with the patients' response.

To further compare the patients' response with the *in vitro* response, TF, CC and M cells were tested for their relative sensitivity to everolimus and sorafenib. TF and M cells had a lower IC50 for everolimus and an equivalent IC50 for sorafenib compared with 786-O cells. Hence, according to our hypothesis, these cells were sensitive to everolimus and sorafenib, a result that paralleled the sensitivity of the first TFE3 patient to both drugs and the transient sensitivity of the second TFE3 patient to everolimus. Our results also suggest that the second TFE3 patient would be sensitive to treatment with sorafenib but this was not possible to verify because the patient died before treatment with third-line chemotherapy. The *in vitro* results suggest that the patient from whom the CC cells were derived was insensitive to everolimus and to sorafenib. The insensitivity to everolimus paralleled the absence of a clear clinical response of the patient who was placed in palliative care. The insensitivity to sorafenib could not be compared with the patient's response since he was not treated with this drug. For this patient, the use of alternative tyrosine kinase inhibitors (axitinib, pazopanib, tivozanib) [27]–[30] or immune strategies through the use of antibodies directed against-programmed death 1 (PD-1)/PD-1 ligand [31]–[32] or against cytotoxic T-lymphocyte-associated protein [33]–[35] would have been a better therapeutic strategy if clinicians had taken into account our *in vitro* results.

**Figure 3:**
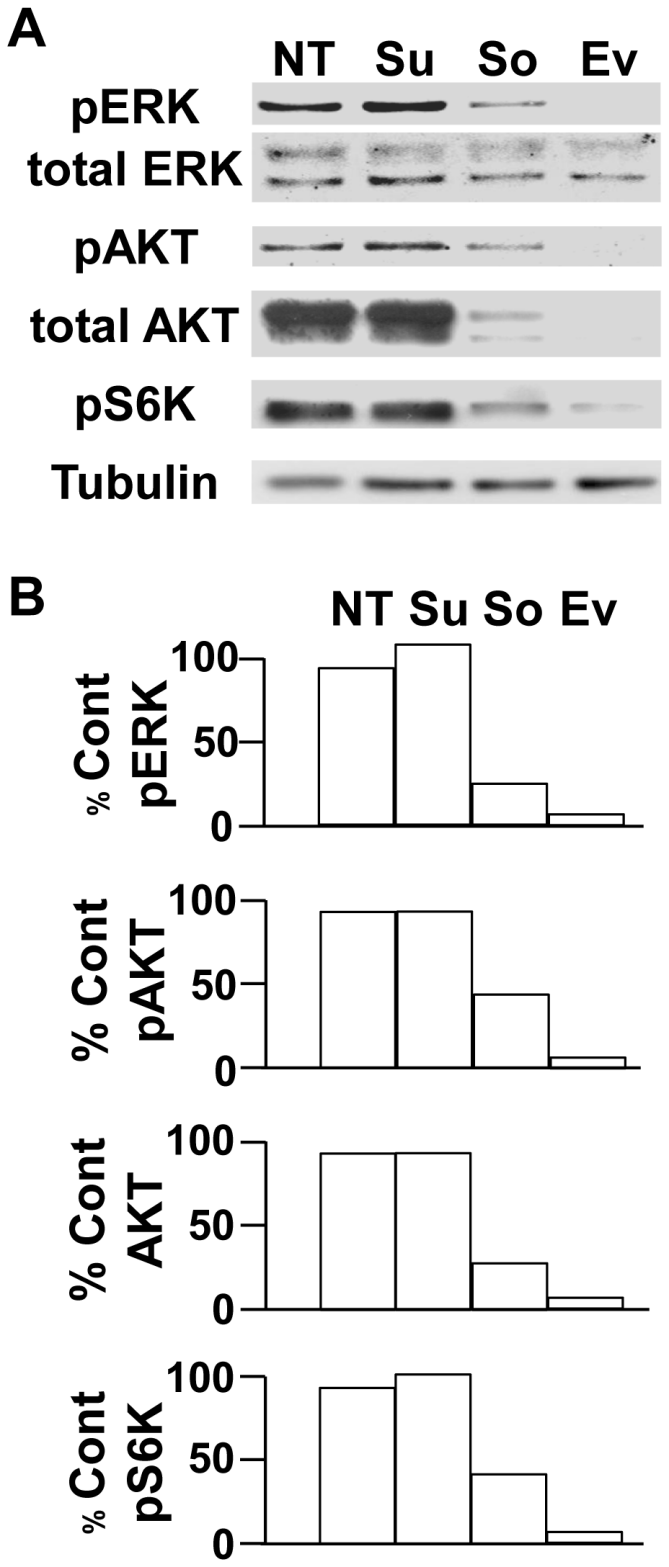
*In vitro* sensitivity of the TF cells to the different drugs used to treat the patient; specific impact on downstream signaling pathways. A) The activity of major signaling pathways (ERK, PI3K/AKT/mTOR/S6K) in response to the different treatments (sunitinib (Su), sorafenib (So) and everolimus (Ev)) was determined by analysis of the presence of the phosphorylated forms of the kinases (pERK, pAKT, pS6K) in TF cells following a short-term treatment (two hours) by immunoblotting. Tubulin is shown as a loading control. B) Quantification of the experiments shown in A. The percentage of phosphorylated kinases or their total amount is indicated with respect to the reference value (100%) for the cell without any inhibitor. These results are representative of four independent experiments.

The effects of the different drugs on proliferation are correlated with the activity of intracellular signaling pathways (ERK and PI3 kinase/AKT/mTOR/S6 kinase (S6K)) in TF cells. Hence, the relative sensitivity of TF cells to the three drugs was shown using western blotting (Fig. 3A and 3B); sorafenib and everolimus inhibited ERK and S6 kinase (S6K) activity but sunitinib did not. Sorafenib and everolimus also induced AKT degradation probably through a proteasome-dependent manner as already suggested [36]–[37]. The effects of the different drugs on signaling pathways have been tested in CC and M cells. Sunitinib strongly inhibited the AKT activity in CC cells without affecting the ERK and S6K activity. Whereas sorafenib slightly inhibited ERK, it potentiated the AKT and S6K activity. Everolimus inhibited S6K with reactivation of AKT. The efficacy of sunitinib and sorafenib observed in the *in vitro* tests on M cells did not correlate with inactivation of ERK and AKT. However, the *in vitro* efficacy of everolimus on the proliferation of M cells may be explained by strong inhibition of S6K (Fig. S1).

**Figure 4:**
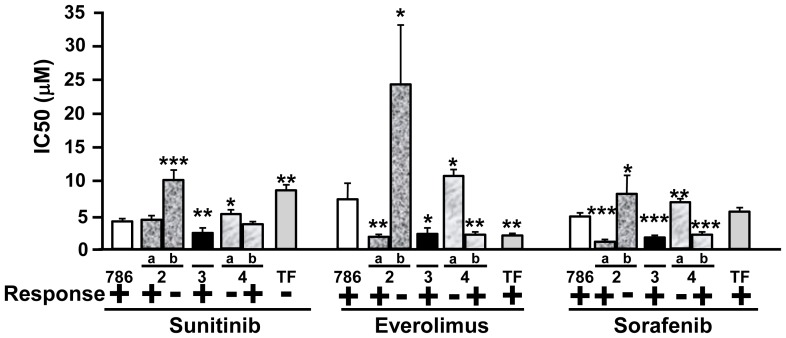
*In vitro* sensitivity of cells isolated from different patients with ccRCC to targeted therapies. Cells were isolated from ccRCC of different Fuhrman grades (two grade 2 tumors (a, b), one grade 3 and two grade 4 (a, b). The IC50 of the different drugs was determined using the MTT test with concentrations of the drugs raning from 0.1 µM to 100 µM and the results were compared with the IC50 in 786-O (786) and TF cells. The expected response of the patients to the different drugs is indicated (− non-responder, + responder). The means, SEMs and p values (Student t test), in comparison with the IC50 for 786-O cells, the reference value, are shown. *p<0.05; **p<0.01; ***p<0.0001.

The sensitivity to the three drugs, which varied from one sample to another, was evaluated in cell samples from five patients with Fuhrman grade 2 to 4 ccRCC, in parallel with 786-O and TF cells (Fig. 4). The relative expression level of the receptors targeted by sunitinib and sorafenib was also investigated and reported in Table 3. Patients 2a, 3 and 4b were expected to be responsive to sunitinib, everolimus and sorafenib whereas patients 2b and 4a were expected to be resistant to the three drugs. For the moment the patients' tumors were not metastatic. Hence, we could not confirm our hypotheses on treatment efficacy. However, our results could be used to inform the choice of treatment in case of progression toward a metastatic grade.

**Table 3:** Level of expression of the different receptors targeted by sunitinib and sorafenib.

	CSFR	FLT3	KIT	PDGFR	RET	VEGFR1	VEGFR2	VEGFR3	Score/8	E Score
786-O	100	100	100	100	100	100	100	100		800
	+	+	−	+	+	−	−	+	5	
2a	679	52	1914	4796	100	1026	15242	30		23839
	+	+	+	+	+	+	+	−	7	
2b	576	38	550	2031	100	281	3162	16		6754
	+	+	+	+	+	+	+	−	7	

(−) indicates that no amplified cDNA was detectable on an agarose gel; (+) indicates amplified cDNA. The *Score/8* indicates the number of receptors targeted by sunitinib and sorafenib. The *E Score* indicates the relative expression level as detected by quantitative and semi-quantitative PCR.

### *In vivo* effects of sunitinib and sorafenib on tumor xenografts obtained with TF cells

**Figure 5:**
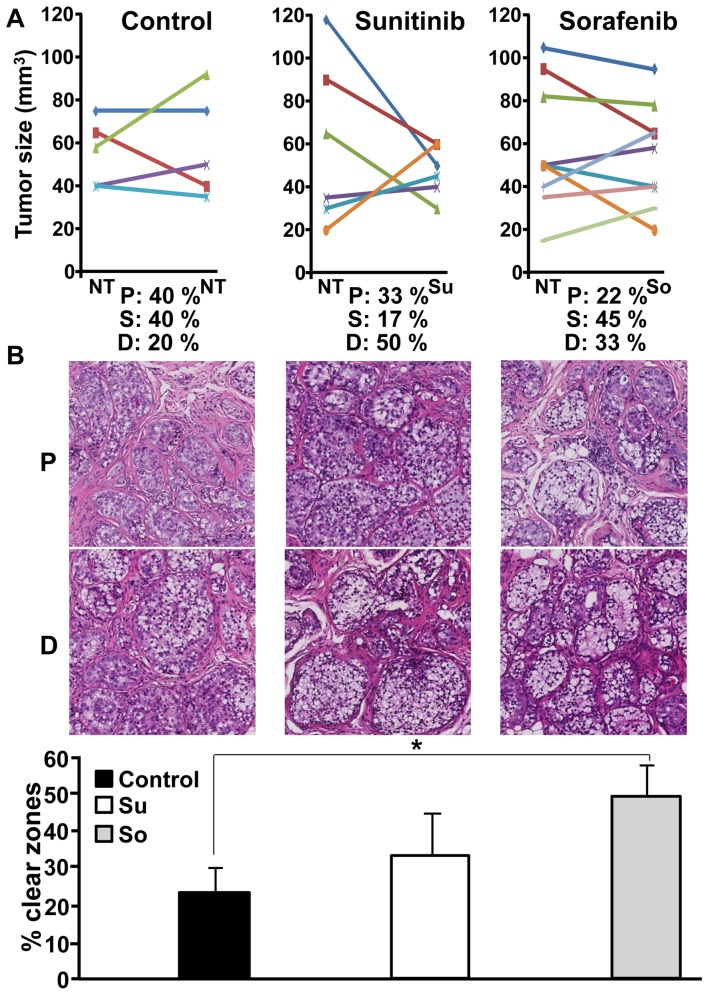
*In vivo* sensitivity of the TF cells to the different drugs used to treat the patient. A) 10^7^ cells were subcutaneously injected into nude mice. When the tumor was visible mice were treated with sunitinib or sorafenib for 15 days. Tumor size was evaluated with a caliper. The percentage of mice with progression (P), decrease (D) or stabilization (S) of the tumor size is indicated. B) The general histological aspect of a progressing tumor (P) or a regressing (D) tumor is shown for the different situations (Control, sunitinib, sorafenib). The percentage of clear zones in stable or responsive tumors was evaluated using the Leica software SlidePath Gateway. Statistical analysis was performed using a student's *t* test (* p<0.05).

The capacity of the drugs to reduce or stabilize tumor growth was tested on subcutaneous xenografts of TFE3 RCC cells in nude mice. The tumor growth was rather long (three months), a result compatible with previous results obtained on direct grafting of tumor fragments of RCC in mice [38]. We considered that a tumor was stable when no modification to the size was observed or if the decrease or increase in size did not exceed 1.5-fold following three weeks of treatment. Without any treatment tumors progressed (40%) or remained stable (40%) (Fig. 5A). One tumor spontaneously decreased in size. Hence, the percentage of tumor that does not progress is 60%. In the presence of sunitinib, only one tumor was stable (17%) and the size of three tumors decreased (50%); the percentage of tumor that does not progress is 67%. In the presence of sorafenib, the growth of most of the tumors was stable (45%). Three tumors regressed (33%); the percentage of tumor that does not progress is 78% which is finally the best score, a situation corresponding to that observed for the patient. Another striking observation was the presence of a majority of cells with clear cytoplasm on HES staining for stable tumors or tumors that regressed during treatment (Fig. 5B). This cytological feature was the same in the initial tumor. Unresponsive or growing tumors had a reduced number of clear zones and in contrast, cells presented an eosinophilic cytoplasm. The percentage of clear zones was evaluated for all tumors (Fig. 5B), only those treated by sorafenib have a significant increase in the number of clear zones.

## Discussion

The advent of targeted therapies has completely revolutionized the treatment of kidney cancers. Until recently, the prognosis was poor in the case of metastatic disease, with a life expectancy of approximately three months. The high level of vascularization defines kidney cancers as a good target for anti-angiogenesis drugs. However, the benefits in terms of global survival are very disappointing. Despite the lack of efficacy in increasing overall survival, the treatments showed, in some patients, a clear benefit on the prolongation of life. In addition to the controversy concerning their efficacy, another parameter to take into account is the price of such compounds, which is very expensive for health insurance companies. The third parameter to consider is related to the side effects associated with the treatments. Having the capacity to stratify patients and to propose to them the best therapeutic option is ideal for the entire health system. To address this question, different parameters have to be considered; i) the capacity to obtain tumor and normal tissue samples of good quality following surgery; ii) the possibility to rapidly establish the sensitivity to the drugs after surgery. When considering the side effects it is essential to define the best option despite the presence of manageable side effects (hypertension, proteinury, hand and foot syndromes); iii) define the best strategy(ies) to demonstrate the most important sensitivity to a given drug. Our experiments complied, at least in part, with these different parameters and provide strong arguments to define the best first line treatment according to *in vitro* results.

Currently, predictive markers of efficacy to anti-angiogenesis drugs are not available in clinical practice. Our results suggest that anti-angiogenesis drug efficacy could be tested in cells derived from the tumor post surgery in order to determine the best first-line treatment. Indeed, these therapies should not to be given to a patient immediately after surgery so that they can completely heal. *In vitro* experiments could be performed during this interval. We showed a correlation between the sensitivity of the patients to the drugs and that of the cells cultured from the primary tumor. The variability in the results obtained in our first experiments was linked to the use of classical DMEM or RPMI media supplemented with fetal bovine serum. The behavior of cells in culture (morphology, proliferation) and the expression of angiogenic factors and cell surface receptors were really different when using the different culture media. The progressive loss of tumor cells expressing the TFE3/NONO translocation was also noted in classical media. The presence of high amounts of target receptors for sunitinib and/or sorafenib in M cells may explain their sensitivity to both drugs. However, despite the presence of target receptors the TF cells were insensitive to sunitinib and CC cells were insensitive to sorafenib suggesting the presence of mutations in the proteins responsible for the activation of downstream signaling pathways (H-RAS/K-RAS, B-RAF/c-RAF and Tuberous Sclerosis Protein 1 [TSC1]) [39]–[40]. However, sequencing of the *H-ras*, *K-ras*, *b-raf*, *c-raf* and *tsc1* genes has not highlighted the presence of specific activating/inactivating mutations. The presence of tyrosine kinase receptors insensitive to sunitinib was also tested. A recent publication has indicated that MET is responsible for the lack of efficacy to anti-angiogenesis treatment in experimental neuroendocrine tumors [41]. Moreover the presence of the co-VEGF receptors neuropilin 1 and 2 (NRP1, 2) participated in cell proliferation and metastasis [42]–[43]. Table S1 shows that whereas MET levels were approximately the same in 786-O and TF cells, the MET ligand hepatocyte growth factor (HGF) was over-expressed in TF cells. NRP1 is also significantly up-regulated in TF cells. These results may explain the higher sensitivity of TF cells and patient to sorafenib (RAF inhibitor), and to everolimus. The higher sensitivity of TF cells to everolimus compared with 786-O cells suggests that they are dependent on the mTOR signaling pathway for proliferation. We also noted that TF cells over-expressed the ELR+CXCL cytokine, CXCL7 (Table S1). This cytokine belongs to a family of pro-inflammatory/pro-angiogenic cytokines whose most studied member is CXCL8/Interleukin 8. They stimulate the ERK and PI3 kinase/AKT/mTOR pathways [44] and induce resistance to sunitinib [23] or bevacizumab [45]. Their receptors, CXCR1/CXCR2, are physiologically expressed on neutrophils/macrophages and endothelial cells but aberrantly expressed by tumor cells, thus creating autocrine proliferation loops [46]–[48]. The presence of such autocrine proliferation loops may explain the insensitivity to sunitinib but sensitivity to sorafenib and everolimus, both drugs inhibit signaling pathways downstream of CXCR receptors. We recently reported that CXCL7 is a marker of poor prognosis for RCC and a new putative therapeutic target [49]. Moreover, the cells isolated from tumors 2a, 3 and 4b were also highly sensitive to everolimus. The relative level of expression of TSC1 in these cells is equivalent to that of 786-O cells. The presence of inactivating mutations is currently being investigated [40]. Cells from tumors 2a, 3 and 4b were also highly sensitive to sorafenib suggesting constitutive activation of RAS and/or RAF. However, the *H-ras* and *b-raf* genes were wild-type in these cells. The relative levels of HGF/MET, NRP1/NRP2 and CXCL cytokines will be tested. Full exome sequencing will be performed on these different cells. Specific attention to mutations of tyrosine kinase receptors that may explain insensitivity to sunitinib and hypersensitivity to inhibitors of down-stream signaling pathways will be made.

**Figure 6:**
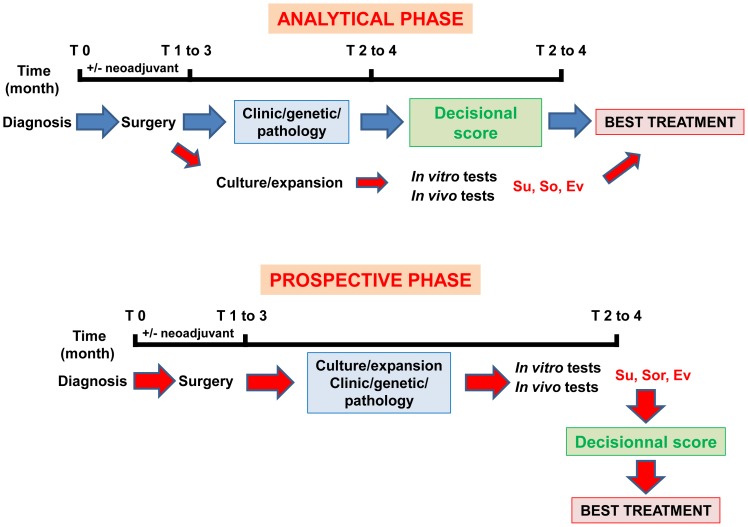
Definition of a standardized method to define the best therapeutic strategy. To validate this method two phases are required. **An ANALYTICAL PHASE**: This phase concerns approximately twenty patients and is dedicated to the definition of an ideal decisional score considering the clinical/pathological and genetic parameters and *in vitro*/*in vivo* experiments. Based on this prospective study on a small number of patients, we observed a strict correlation between the cell and the patient response to a given drug with a success rate of 100% for our methodology, which aims at the determination of the most efficient treatment. If a strong correlation exists between our score and the success to the treatment a **PROSPECTIVE PHASE** will be envisaged. For this trial, treatments will be administered according to the best decisional score.

Our *in vitro* experiments were supplemented by an *in vivo* approach using xenografts of TF cells in nude mice. Even though the relative resistance to sunitinib and sensitivity to sorafenib were evidenced, tumor development in nude mice is long and not compatible for the choice of the first-line therapy [38]–[50]. Moreover, some mouse tumors generated with cells that we considered resistant present sensitivity to sunitinib. These results are consistent with the fact that patients who progressed on sunitinib and who were treated with others drugs for a while and then became progressively resistant to the different therapeutic lines, may be sensitive again to sunitinib [51]. These probably highlight the tumor cell population heterogeneity [52]. Hence, the *in vitro* general response is proportional to the percentage of sensitive and resistant cells and finally more relevant than the *in vivo* response in mice. Finally, the selection pressure exerted by the treatment favors the emergence of resistant cells already present within the tumor or in the cell population derived from the tumor. Our methodology should now be evaluated in a larger prospective series of patients. Hence, our promising results will initiate a clinical trial with recruitment of patients from March 2014. A general plan for the evaluation of this protocol and its impact on clinical decision-making is shown in Fig. 6. In theory, this methodology could be applied to any type of tumor for which the sensitivity to targeted therapies should be evaluated.

## Supporting Information

Figure S1 ***In vitro*** **sensitivity of the CC and M cells to the different drugs used to treat the patients; specific impact on downstream signaling pathways.** A) The activity of major signaling pathways (ERK, PI3K/AKT/mTOR/S6K) in response to the different treatments (sunitinib (Su), sorafenib (So) and everolimus (Ev)) was determined by analysis of the presence of the phosphorylated forms of the kinases (pERK, pAKT, pS6K) in TF cells following a short-term treatment (two hours) by immunoblotting. Tubulin is shown as a loading control. B) Quantification of the experiments shown in A. The percentage of phosphorylated kinases or their total amount is indicated with respect to the reference value (100%) for the cell without any inhibitor. These results are representative of four independent experiments.
(TIF)

Table S1 (DOCX)
