# Supplementary figures and images for: The Relevance of Testing the Efficacy of Anti-Angiogenesis Treatments on Cells Derived from Primary Tumors: A New Method for the Personalized Treatment of Renal Cell Carcinoma
Source: PLoS One. 2014 Mar 27;9(3):e89449. doi: 10.1371/journal.pone.0089449 (PMC3968004; doi:10.1371/journal.pone.0089449)

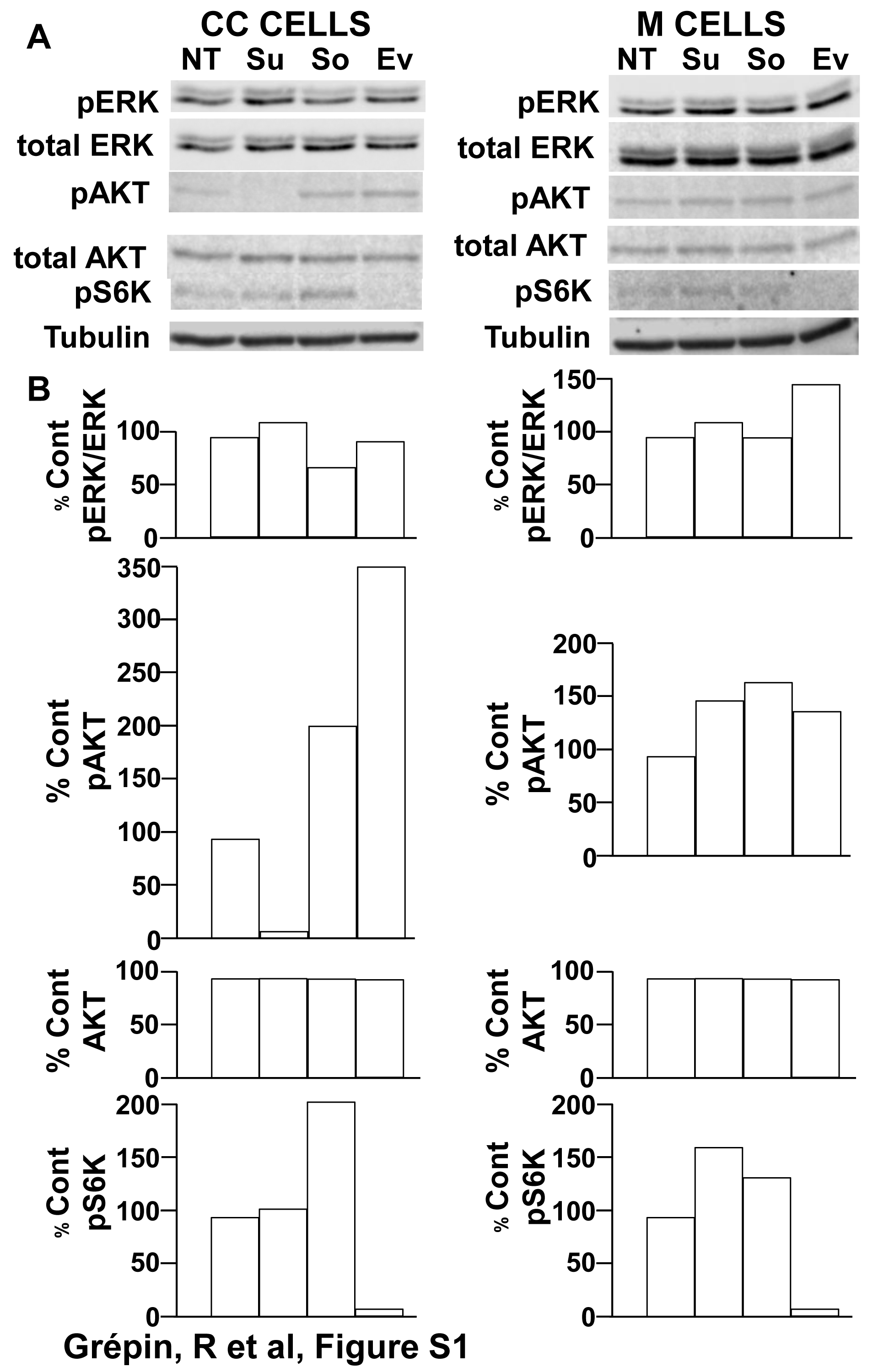

Supplement: Figure S1 — In vitro sensitivity of the CC and M cells to the different drugs used to treat the patients; specific impact on downstream signaling pathways. A) The activity of major signaling pathways (ERK, PI3K/AKT/mTOR/S6K) in response to the different treatments (sunitinib (Su), sorafenib (So) and everolimus (Ev)) was determined by analysis of the presence of the phosphorylated forms of the kinases (pERK, pAKT, pS6K) in TF cells following a short-term treatment (two hours) by immunoblotting. Tubulin is shown as a loading control. B) Quantification of the experiments shown in A. The percentage of phosphorylated kinases or their total amount is indicated with respect to the reference value (100%) for the cell without any inhibitor. These results are representative of four independent experiments. (TIF) [file pone.0089449.s001.tif]
