# Supplementary material for: The Relevance of Testing the Efficacy of Anti-Angiogenesis Treatments on Cells Derived from Primary Tumors: A New Method for the Personalized Treatment of Renal Cell Carcinoma
Source: PLoS One. 2014 Mar 27;9(3):e89449. doi: 10.1371/journal.pone.0089449 (PMC3968004; doi:10.1371/journal.pone.0089449)
Supplement: Table S1 — (DOCX) [file pone.0089449.s002.docx]

**Table S1: Level of expression of the different genes putatively implicated in sunitinib inefficacy.**

The percentage expression of the different cytokines and receptors is shown. The reference values (100%) correspond to the levels of the sensitive 786-O cells. Only statistically significant values are shown.
